# Mirror Therapy Rehabilitation in Stroke: A Scoping Review of Upper Limb Recovery and Brain Activities

**DOI:** 10.1155/2021/9487319

**Published:** 2021-12-31

**Authors:** Nurulhuda Jaafar, Ahmad Zamir Che Daud, Nor Faridah Ahmad Roslan, Wahidah Mansor

**Affiliations:** ^1^Centre for Occupational Therapy Studies, Faculty of Health Sciences, Universiti Teknologi MARA, Puncak Alam, Selangor, Malaysia; ^2^Department of Rehabilitation Medicine, Faculty of Medicine, Universiti Teknologi MARA, Sungai Buloh, Selangor, Malaysia; ^3^Microwave Research Institute, Universiti Teknologi MARA, Shah Alam, Selangor, Malaysia; ^4^School of Electrical Engineering, College of Engineering, UiTM Shah Alam, Malaysia; ^5^Computational Intelligence Detection, Health & Wellness ReNeU, UiTM Shah Alam, Malaysia

## Abstract

**Background:**

Mirror therapy (MT) has been used as a treatment for various neurological disorders. Recent application of electroencephalogram (EEG) to the MT study allows researchers to gain insight into the changes in brain activity during the therapy.

**Objective:**

This scoping review is aimed at mapping existing evidence and identifying knowledge gaps about the effects of MT on upper limb recovery and its application for individuals with chronic stroke.

**Methods and Materials:**

A scoping review through a systematic literature search was conducted using PubMed, CINAHL, PsycINFO, and Scopus databases. Twenty articles published between 2010 and 2020 met the inclusion criteria. The efficacy of MT on upper limb recovery and brain activity during MT were discussed according to the International Classification of Functioning, Disability and Health (ICF).

**Results:**

A majority of the studies indicated positive effects of MT on upper limb recovery from the body structure/functional domain. All studies used EEG to indicate brain activation during MT.

**Conclusion:**

MT is a promising intervention for improving upper limb function for individuals with chronic stroke. This review also highlights the need to incorporate EEG into the MT study to capture brain activity and understand the mechanism underlying the therapy.

## 1. Introduction

Stroke is the second largest cause of early death and secondary disabilities [1]. Motor skills are among the crucial areas affected by stroke, and recovery from stroke typically takes more than six months, especially in the upper limbs. Evidence shows that about 83% of stroke survivors are able to walk again; however, only 5% to 20% of survivors achieve full functional recovery of affected upper limbs [2].

Several therapies using different technological sophistication levels have emerged to restore motor function after stroke. Dr. Ramachandran introduced mirror therapy (MT) in the 1990s to manage numerous other conditions, including motor disorders [3]. This therapy is based on visual stimulation. Visual feedback is given to the individual using a mirror that reflects the nonaffected limb to make the brain believe that what the individual sees is the affected limb moving without difficulty [4]. Unilateral and bilateral procedures have been implemented since the introduction of MT. In the unilateral procedure, activities are performed only on the unaffected limb. In the bilateral procedure, the individual attempts to move the affected limb as much as possible to mimic the reflected movements of the unaffected limb. Although there are different variations in MT setup and procedure, all methods serve to aid the recovery of the affected limb by stimulating the regions of the brain associated with movement, sensation, and pain [5]. The literature suggests that recovery between the first three to six months of onset is largely natural [6]. Recovery in the stroke context is defined as restoring the ability to perform a movement in the same way as before the injury [7].

The selection and classification of outcomes in stroke rehabilitation are primarily based on the International Classification of Functioning, Disability and Health (ICF) framework. The domains in the ICF include human functioning, which comprises body structure/function, activity, and participation. In any intervention study focusing on the stroke population, it may be crucial for scholars to address the effects of the interventions on the body structure/function domain. However, it is equally crucial to examine the effects of these changes on individual activity and participation domains [8].

Advanced electroencephalogram (EEG) technology for analysing brainwave signals has brought a new perspective in stroke research by capturing meaningful electrophysiological features of neuron activities [9]. Predicting therapy outcomes is more difficult in the chronic phase where the duration since stroke onset is six months or more. This is because motor recovery is not necessarily linked to the degree of the initial injury. Several complex mechanisms of dynamic neuroplasticity occurs after the initial stroke lesion [10]. Therefore, predicting motor function requires the use of complementary techniques. EEG is one of the neurophysiological techniques that can provide helpful information for predicting clinical outcomes. EEG is not only used for “for predicting clinical outcomes.” Evidence also suggests that EEG may provide insightful information on neural activity changes and interhemispheric differences [11, 12]. It is presently feasible for researchers to objectively evaluate changes in brain activity before and after the intervention. The MT study continues to evolve with various protocols and different targeted populations. The previous MT reviews have focused on all stroke phases [5, 13]; however, there are limited studies that emphasised the changes in brain activity after MT for individuals having chronic stroke only. Therefore, the purpose of this review is to map existing evidence and knowledge gaps concerning MT on brain activity and upper limb recovery among individuals with chronic stroke.

## 2. Methods

### 2.1. Design

A scoping review offers an opportunity to broadly map and summarise existing research findings and identify gaps in existing knowledge. It is the most appropriate methodology to achieve the objective of mapping brain activity using EEG. There is a wide range of methodologies and protocols in the MT study for which scoping reviews are the recommended methodology. The framework outlined by Arksey and O'Malley [14] is used for this review and involved identifying the research question; identifying relevant studies and study selection; charting the data; and collating, summarising, and reporting the results. This review also relies on the PRISMA-ScR checklist to ensure that the scoping review is robust and includes all essential reporting items [15].

### 2.2. Information Sources and Search Strategy

The search was limited to publications between 2010 and 2020. It was conducted using four electronic databases, namely, PubMed, CINAHL, PsycINFO, and Scopus for relevant articles that contain keywords in the title, abstracts, or words including “mirror therapy,” “mirror visual feedback,” “mirror feedback,” “mirror box therapy,” “mirror training,” “mirror illusion,” Stroke, “cerebrovascular accident,” cva, hemiplegia, electroencephalogram, EEG, “brain waves,” “hand function,” “upper limb recovery,” “motor function,” “upper limb function,” “motor recovery,” or “motor performance.” Boolean operators, truncations, wildcards, and MeSH terms related to these keywords were used whenever possible. The keywords were inserted into electronic search engines on May 2020 and were updated up to November 2020.

### 2.3. Eligibility Criteria

All retrieved articles were screened for relevance based on titles and abstracts. The studies were included if they (i) consisted of conventional mirror therapy interventions in one of the treatment groups; (ii) reported at least one outcome related to upper limb recovery or brain activity using EEG; (iii) presented level II-IV evidence according to CEBM levels of evidence, in which the study types included in this scoping review are all types of original or primary interventional studies (i.e., randomized controlled trials, quasiexperiments, and single-group pre-post) and observational studies (i.e., cross-sectional, cohort, longitudinal, case series, and case reports); (iv) were published between 2010 and 2020; and (v) comprised a full-text peer-reviewed article published in English. Studies were excluded if they (i) were not targeted at adults with stroke duration of six months and above.

### 2.4. Selection of Sources of Evidence

Four databases are chosen based on the review topic (medical and health science research) and access to the databases. All citations retrieved from the databases were uploaded into Mendeley after removing duplicates. All authors independently reviewed titles and abstracts against the selection criteria. The remaining citations were then reviewed as full-text articles for inclusion.

### 2.5. Data Charting Process

Data were extracted from included articles, entered into a Microsoft Excel spreadsheet by the first and last authors, and reviewed by other authors. The following data items were extracted: title, sample size, study design, outcome used, type of intervention, and findings.

## 3. Results

**Figure 1:**
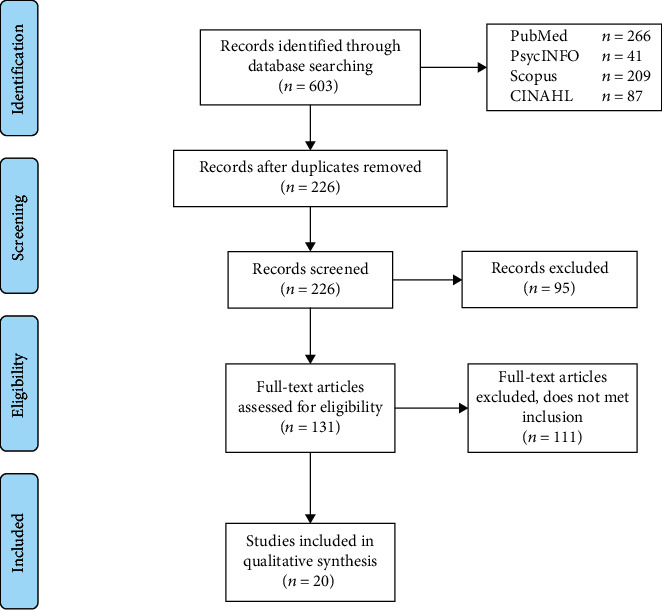
Flow diagram outlining the search and study selection.

Results of the systematic search and review process are presented in Figure 1. The search produced a total of 603 abstracts across the four databases. After removing 377 duplicates, 226 original abstracts were screened, and full texts were evaluated based on the inclusion criteria. Twenty studies met the inclusion criteria and were included for data extraction and synthesis. Seventeen studies related to MT for upper limb recovery while three studies related to MT with brain activity using EEG.  Table 1 shows details of the 17 studies included in this review for upper limb recovery and changes in brain activity following MT. Fourteen studies are based on randomized controlled trial (RCT) design, two studies are based on quasiexperimental design, and one study uses a case-control design. The number of subjects ranged between 4 and 120.  Table 2 shows the details of three studies concerning brain activity. Two studies are quasiexperimental, while the other is a longitudinal study. The number of subjects ranged between 1 and 20.

### 3.1. Outcome Measures across ICF Domain Used in MT Studies

The ICF model provides a framework for classifying outcomes, which is a critical aspect of clinical research [16]. Integration of the ICF model into MT research and practice would provide a foundation for a common language, particularly in examining MT outcomes. More than ten upper limb outcome measures were used in all the studies. Fifteen studies [17–31] used Fugl-Meyer Assessment (FMA) for body structure/function domain. Four studies investigated sensory function using Nottingham Sensory Assessment (NSA) and Semmes Weinstein monofilament test [17, 19–21]. Six out of thirteen studies [15, 21, 23, 25, 27, 28] used the Box and Block Test (BBT) for activity domain outcome measures, and six studies [15, 21–24, 28] used Functional Independence Measure (FIM). Three studies measured the activity domain using the ABILHAND measure and Motor Activity Log (MAL) and found that MT had no significant effect on these measures [16, 26, 27].

### 3.2. Effectiveness of Conventional Mirror Therapy

There are seventeen articles concerning MT study that evaluated the outcomes of upper limb function. Nine studies compared the MT group with conventional therapy [14, 16, 17, 20, 23, 24, 27, 29, 30] and five studies with sham therapy [15, 21, 22, 25, 31]. One study [17] used passive mobilisation, while another study [30] used bilateral training for the control group. In MT groups, eight studies each reported using the unilateral and bilateral procedures. Only one study used both unilateral and bilateral procedures. In the body structure/impairment domain, seven out of the eight [13, 14, 21–25] using the unilateral procedure had positive findings favouring the MT group against conventional therapy. For the bilateral procedure, six out of eight studies [16, 26, 27, 30–32] showed significant differences between the two groups favouring the bilateral MT procedure. One study [21] used both procedures during MT and used the body structure/function domain outcome measure only for cases where MT group improvement was significant compared to conventional therapy. Two unilateral and one bilateral procedure studies did not include outcome measures in the activity domain. Among studies that used the unilateral procedure, two out of six studies did not show significant results favouring the MT group [13, 22] in the activity domain. In contrast, only two of seven bilateral MT procedure studies [30, 32] indicated significant improvements favouring MT.

### 3.3. MT Protocol

The total duration of the therapy program varied between four to eight weeks, with the frequency of intervention varying between three and five times per week. The number of sessions ranged between twelve and forty, where eight out of seventeen studies had twenty sessions in total. Mirror exposure hours varied between forty-five minutes and five hours per week. Ten out of seventeen studies comprised less than three hours per week of mirror exposure [13, 20–25, 30–32]. Among the ten studies, eight reported positive effects on upper limb measures favouring the MT group. For MT exercise, nine studies [13, 15, 16, 24, 26–28, 30, 32] used simple exercises and functional tasks during MT sessions. Five studies [20–22, 25, 31] used simple exercises, and three studies used functional tasks. Only seven studies mentioned mirror size, with height ranging between 30 cm and 46 cm, and length ranging between 31 cm and 61 cm.

### 3.4. Brainwave Activity

Table 2 shows three articles included in this review, where brain activity is assessed using EEG when MT was applied to individuals with chronic stroke. Two studies studied four weeks of MT. Only one article [23] studied the long-term effects of MT on brain activity. It is a longitudinal study conducted over nine months. All articles showed changes in brain activity in response to MT. Considering the five rhythms included in this study, researchers found that the most consistent and significant MT effect occurred for the mu rhythm. One article examined mu rhythm brainwaves [23]; another study [33] used alpha and beta waves. In contrast, one study does not mention any waves [22]. Chang et al. found a higher degree of alpha waves when the individual observed the hand, while beta waves were higher when the individual observed the mirror reflection [33]. Lee et al. do not mention any waves but revealed that brain activity increases after MT interventions. They performed brain mapping by visually examining changes in the brain regions during MT [22]. Chang et al. [33] used six EEG channels, C3, C4, F3, F4, O1, and O2, while Rosipal et al. [23] used twelve EEG channels C1-C6, FC3, FC4, CP3, CP4, O1, and O2 in the studies.

## 4. Discussion

This scoping review is aimed at mapping existing evidence and knowledge gap about the effects of MT on upper limb recovery and brain activity using EEG among individuals with chronic stroke. The majority of the studies showed positive effects of MT on upper limb recovery. In stroke rehabilitation, the proportional recovery rule is widely accepted. There is a “critical window for recovery” within the first 3–6 months poststroke, and this duration enhanced neuroplasticity mechanisms triggered by the injury [34]. However, a study showed a long-lasting critical period of poststroke enhanced neuroplasticity that enables improvement in body function and structure even at chronic stages. Some people demonstrate improvement over 24 months after the stroke [35]. Therefore, in line with this review, recovery is not limited to the golden period (3-6 months); it is still relevant for chronic stroke phases when MT is performed.

This review suggested that the use of MT as an adjunct to conventional methods provides additional benefits for upper limb functional recovery. However, it can be concluded that there are inconsistent results concerning improvement in the activity domain. Individuals with chronic stroke eventually adjust or compensate for their impairment where they often do not involve or use their affected limb in an at-home setting. Consequently, therapeutic improvements deteriorate rapidly, and improvement is not seen in the activity domain outcome measures [30]. The majority of studies applied less than three hours per week of mirror exposure. There is no difference in the positive results between the procedures that applied more or less than three hours of mirror exposure per week. In contrast, a study by Palaskar [36] suggests that a high-intensity mirror therapy program may be more beneficial in rehabilitating hand functions where five hours per week of therapy is provided instead of two-and-half hours per week during the acute stroke phase.

According to a previous study, there are three hypotheses concerning the underlying mechanism of MT [37]. First, MT is thought to activate the mirror neuron system (MNS). Mirror neurons fire when an individual observes an action and then performs a similar action. Second, it might promote the recruitment of ipsilateral motor pathways. Third, paying attention to the affected limb may activate motor networks when individuals observe an illusory image of a “healed” paretic limb. No concrete conclusion could be drawn between unilateral and bilateral procedures. Both methods showed significant improvement in upper limb recovery specific to body structure/function domain outcome measure.

Bilateral exercise training without a mirror includes the recruitment of the ipsilateral corticospinal pathways, normalisation of inhibitory mechanisms, and increased control of the contralesional hemisphere [38]. A bilateral MT procedure is suggested to have a similar underlying mechanism. On the contrary, unilateral therapy might be related to the first hypothesis and more influenced by the activation of the mirror neuron system (movement observation) than corticospinal pathways. Therefore, both methods may provide positive effects using different mechanisms. Stroke survivors exhibit relatively diminished poststroke mu suppression over the affected hemisphere.

MT embedded in long-term rehabilitation could provide additional neurophysiological benefits to the individuals. More evidence of mu suppression indicates that mirror neuron system activities can be increased through MT [9]. However, there are still limited studies that used EEG for identifying mu suppression during MT sessions. Michielsen and colleagues [30] used fMRI to identify changes in the activation balance within primary motor cortex of the affected hemisphere in the MT group immediately following intervention, which is related to a second hypothesis. MT can contribute to a shift in activation toward the affected lesion, inducing more symmetrical activity between the two hemispheres.

All studies that used EEG showed MT activated brain activity in individuals with chronic stroke. Three brainwaves were identified related to MT interventions: alpha, beta, and mu rhythm. When relaxed, alpha waves increase by about three-fourths. While working on tasks requiring more attention, alpha waves were inhibited while beta waves increased [39]. Beta waves increase during mirror observation, indicating that the premotor and prefrontal cortex are activated because the subjects paid more attention to the movement during this condition.

Recently, changes in the strength of the mu frequency band (mu power) have been used to study MNS, where mu suppression may be a specific index for mirror neuron activities. The introduction of standardised caps made it easier to routinely identify mu rhythms from EEG sites C3 and C4 (central sites situated over the sensorimotor cortex) [40]. All the articles in this review used C3 and C4 channels. In stroke rehabilitation, Rahim et al. [41] proposed four channels, C3, C4, F3, and F4, to monitor motor recovery status since these channels are associated with motor planning and sensory-motor integration. Bae et al. [42] found that mu rhythm suppression was significant after the mirror therapy treatment for both groups, with a better result in the MT group.

FMA and BBT were the most frequently used outcome measures to investigate the effectiveness of MT in improving upper limb function recovery. FMA is a stroke-specific and performance-based impairment index for poststroke recovery. It is commonly used to evaluate the recovery at all stroke stages [43]. FMA-UE results are the most accepted measure for body structure/function domain [44]. This assessment has excellent intrarater and interrater reliability [45, 46]. BBT is aimed at evaluating manual dexterity, which has excellent test-retest, inter-, and intra-rater reliability [47]. The results in the activity domain are inconsistent, where only a few studies showed significant effects between groups [13, 16, 22, 26]. One reason is that some tools used to measure the domain, such as ABILHAND and Motor Activity Log (MAL), are semistructured interview-based assessments instead of performance-based assessments. The user or clinician should note that self-estimated measures are subject to overestimation or underestimation of actual performance when scores are not based on clinician performance observation [48]. Individual factors such as self-esteem, insight, vision, language, and cognitive function prior to administering the assessment should be considered. Another reason for the inconsistency of results in the activity domain may be due to the ordinal measurement of the outcomes that usually use Likert scale to be rated by respondents [49]. The distances between scores in an ordinal scale are separated by unknown quantities of measured variable, so the unit distance between adjacent categories can vary in meaning across the scale [50]. When ordinal scales are used, this clinimetric behaviour may result in misleading findings on measurement of change and responsiveness [50]. Nowadays, scholars suggest Rasch analysis to be used broadly to overcome this issue. Rasch modelling of ordinal data allows for the transformation of ordinal raw scores into interval scale measures under certain conditions and within a probabilistic framework [51].

No studies reported participation outcome. This is most likely because of the diverse definitions and interpretations of participation as a concept and some of the outcomes on participation referred to mobility, fitness and emotion [52, 53] There is also a wide variety of tools purporting to measure participation making it challenging and difficult to be interpreted. Moreover, there are many extraneous factors that influence both participation and quality of life [54]. Previous studies revealed that there is a positive relationship between participation and environmental factors, including features of the natural and built world, as well as attitudes of others and social policies that affect people with disabilities [55]. Therefore, it is difficult to incorporate participation when the mirror therapy intervention for upper limb motor functions is provided in the clinic environment that has limited resources.

This study should be viewed as a map for future research. There are knowledge gaps in the MT procedure and protocol. Therefore, this scoping warrants a future study comparing mirror procedures (unilateral and bilateral) to know which procedure is best for individuals with chronic stroke. EEG has recently been used for detecting neuroplasticity because of its excellent temporal resolution and high sensitivity to human brain activity fluctuations [56]. However, EEG findings in MT studies are still limited and require further investigation to explain the underlying mechanism and advance the understanding of brain activity during MT. EEG is a potential and straightforward tool to prognosticate the intervention outcome and facilitate meaningful therapy for individuals with stroke.

## 5. Conclusion

In conclusion, MT is a promising intervention for improving upper limb recovery and brain activity. MT may be considered for rehabilitation programs for individuals with chronic stroke to facilitate better outcomes. Using advanced functional neuroimaging and electrophysiological approaches such as EEG, researchers can now objectively evaluate brain activity before and after the intervention. The damaged regions of the brain must be reorganized to improve upper limb recovery. Hence, EEG can provide useful information on brain activity when MT is clinically applied to individuals with stroke. Because of its practicality, MT is a very appealing technique for stroke rehabilitation to improve upper limb functional recovery and induce brain neuroplasticity.

## Figures and Tables

**Table 1:** The summary of reviewed articles that used at least one outcome of upper limb recovery.

Author (year)	Sample size	Subjects' characteristics	Study design	MT group	Control intervention	MT protocol				Outcome measures		Findings
						Total sessions	Hours of exposure to mirror/week	Type of exercise	Size of mirror (cm)	Body structure/function	Activity	
Colomer et al. (2016) [17]	31	Age: 53.8 ± 5.5 Stroke types: Ischemic and haemorrhagic Severe paresis upper limb Brunnstrom stages I or II FMA below 19	RCT	UMT 45 mins, 3x/week, 8 weeks	Passive mobilisation 45 mins, 3x/week, 8 weeks	24	2 hrs 15 mins	Simple exercise Functional task	Not stated	FMA NSA	WMFT	MT group provided a similar motor improvement
Choi et al. (2019) [57]	36	Age: 59.58 ± 11.87 Stroke types: Not stated MAL below 2.5	RCT	(1) BMT (2) GR+MT 30 mins, 3x/week, 5 weeks	ST 30 mins, 3x/week, 5 weeks	15	1 hr 30 mins	Simple exercise	Not stated	MFT	—	The difference between conventional MT and CG significant in MFT
Arya et al. (2015) [18]	33	Age: 48.76 ± 13.58 Stroke types: Ischemic and haemorrhagic Brunnstrom 2 or above	Pilot RCT	UMT 90 mins, 5x/week, 8 weeks	CT 90 mins, 5x/week, 8 weeks	40	3 hrs 45 mins	Functional task	61 × 46 × 36	FMA	—	MT group exhibited highly significant improvement on FMA scores
Guo et al. (2019) [24]	120	Age: 67.15 ± 11.23 Stroke types: Ischemic and haemorrhagic MAS 2-3	RCT	(1) UMT (2) ESWT+MT 50 mins, 5x/week, 4 weeks	(1) ESWT (2) CT 30 mins, 5x/week, 4 weeks	20	1 hr 40 mins	Simple exercise	30 × 30	FMA	—	MT combined with ESWT produced greater improvement in upper extremity motor performance than MT alone
Arya et al. (2018) [21]	31	Age: 44.12 ± 9.08 Stroke types: Ischemic and haemorrhagic Diminished light touch	RCT	UMT and BMT 90 mins, 5x/week, 6 weeks	CT 90 mins, 5x/week, 6 weeks	30	3 hrs 20 mins	Simple exercise/functional task	61 × 46 × 36	FMA Semmes Weinstein monofilament	—	FMA scores significantly increase in the MT group compared to control
Park et al. (2015) [25]	30	Age: 56.2 ± 13.4 Stroke types: Ischemic and haemorrhagic Brunnstrom IV	RCT	UMT 30 mins, 5x/week, 4 weeks	ST 30 mins, 5x/week, 4 weeks	20	2 hrs 30 mins	Simple exercise	Not stated	FMA	BBT FIM	MT group significantly improved on upper-extremity function and activities of daily living compared to CG
Michielsen et al. (2011) [30]	40	Age: 55.3 ± 12.0 Stroke types: Ischemic and haemorrhagic Brunnstrom III and IV	RCT	BMT 60 mins, 5x/week, 6 weeks	Bilateral training 60 mins, 5x/week, 6 weeks	30	5 hrs	Simple exercise Functional task	Not stated	FMA	ARAT ABILHAND	FMA improved more in the MT group than CG. No sig. difference in ARAT and ABILHAND
Gurbuz et al. (2016) [26]	31	Age: 60.9 ± 10.9 Stroke types: Ischemic and haemorrhagic Hospitalised Brunnstrom I-IV	RCT	UMT 20 mins, 5x/week, 4 weeks	ST 20 mins, 5x/week, 4 weeks	20	1 hr 40 mins	Simple exercise	Not stated	FMA	FIM	FMA score higher in the MT group than CG No sig. difference between groups for FIM
Lin et al. (2014) [31]	43	Age: 56.01 ± 12.53 Stroke types: Ischemic and haemorrhagic Brunnstrom III or above	RCT	(1) BMT (2) MG+MT 90 mins, 5x/week, 4 weeks	CT 90 mins, 5x/week, 4 weeks	20	5 hrs	Simple exercise Functional task	Not stated	FMA	BBT MAL ABILHAND	MT + MG and MT groups performed better than CG in the reduction of motor impairment Combining MT+MG stimulation showed additional effects on manual dexterity of the affected hand compared with MT alone No significant different in MAL and ABILHAND
Oliviera et al. (2018) [58]	21	Age: 60.1 Stroke types: Not stated	Pilot quasi experimental	BMT 15 mins, 3x/week, 4 weeks	(1) VG (2) CT 15 mins, 3x/week, 4 weeks	12	1 hr	Simple exercise Functional task	Not stated	Rivermead Mobility Index	WMFT JHFT	Significant findings were observed for MT or VT group when compared to the CG, obtaining improvements in all three functional tests
Lee et al. (2015) [19]	48	Age: 56.64 ± 9.43 Stroke types: Not stated Moderate-mild impairment (FMA:18-55)	RCT	(1) BMT (2) MG+MT (3) Sham MG+MT 90 mins, 5x/week, 4 weeks	—	20	5 hrs	Simple exercise Functional task	41 × 50 × 33	FMA rNSA	BBT FIM	No significant group differences in the FMA, rNSA. For BBT and FIM, MT+MG improved more than MT group
Kim et al. (2016) [27]	25	Age: 45.2 ± 4.7 Stroke type: Ischemic and haemorrhagic	RCT	UMT 30 mins, 5x/week, 4 weeks	CT 30 mins, 5x/week, 4 weeks	20	2 hrs 30 mins	Functional task	46 × 61	FMA	ARAT BBT FIM	MT group showed significant improvements compared to CG, both in body structure/function and activity domain
Lin et al. (2014) [32]	16	Age: 55.64 Stroke type: Ischemic and haemorrhagic Brunnstrom >III	RCT: pilot study	(1) BMT (2) MG+MT 90 mins, 5x/week, 4 weeks		20	5 hrs	Simple exercise Functional task	Not stated	MAS	BBT ARAT FIM	BBT, grasping scales ARAT, FIM presented significantly large effects in favour of MT+MG group
Shaker et al. (2020) [59]	30	Age: 49 ± 8.56 Stroke types: Ischemic MMT: Grade 3 above	Case control	BMT 40 mins 3x/week, 8 weeks	CT 40 mins, 3x/week, 8 weeks	24	1 hr 15 mins	Simple exercise Functional task	35×35	ROM (goniometer) Strength (dynamometer)	JHFT	MT group improved significantly in ROM, hand strength and JHFT compared to CG
Chinnavan et al. (2020) [28]	25	Age: 45 to 65 years old Stroke types: Ischemic and haemorrhagic	Quasi experimental	UMT 45 mins, 3x/weeks, 6 weeks	CT 45 mins, 3x/week, 6 weeks	18	45 mins	Simple exercise Functional task	Not stated	FMA	FIM	There is significant improvement in MT group compared to CG in both domains.
Ji et al. (2014) [29]	35	Age: 50.53 ± 8.02 Stroke types: Ischemic and haemorrhagic	RCT	(1) UMT (2) rTMS+MT 30 mins, 5x/week, 6 weeks	ST 30 mins, 5x/week, 6 weeks	30	2 hrs 30 mins	Simple exercise	35 × 35	FMA	BBT	MT+rTMS more effective to improve upper extremity function, than MT group and CG
Wu et al. (2013) [20]	33	Age: 54.77 ± 11.66 Stroke types: Ischemic and haemorrhagic Mild to moderate impairment (FMA: 26-56)	RCT	BMT 90 mins, 5x/week, 4 weeks	CT 90 mins, 5x/week, 4 weeks	20	5 hrs	Simple exercise Functional task	Not stated	FMA rNSA	MAL ABILHAND	FMA showed sig. and large to moderate effects favouring the MT group. No sig. differences on MAL and ABILHAND

UMT: unilateral mirror therapy; BMT: bilateral mirror therapy; CT: conventional therapy; ST: sham therapy; CG: control group; FMA: Fugl-Meyer Assessment; ARAT: Action Research Arm Test; WMFT: Wolf Motor Function Test; BBT: Box and Block Test; rNSA: revised Nottingham Sensory Assessment; MAL: Motor Activity Log; JHFT: Jebsen Hand Function Test; FIM: Functional Independence Measure; RCT: randomized controlled trial; rTMS: transcranial magnetic stimulation; MG: mesh glove; GR: gesture recognition; ESWT: extracorporeal shockwave therapy; VG: vibration.

**Table 2:** The summary of the articles using EEG.

	Sample size	Subjects' characteristics	Study design	MT groups	Control groups	Outcome measures	Findings
Chang et al. [33]	14	Chronic	Quasi experimental	5x/week, 4 weeks	No control group	EEG	Alpha power higher in hand observation compared to mirror observation in F3, F4, O1, and O2 channels
Lee and Han [22]	2	Chronic	Quasi experimental	30 mins, 5x/week, 4 weeks	No control group	EEG	The brain activity increased after treatment
Rosipal et al. [23]	1	Chronic	Longitudinal	2x/week, 9 months	No control group	EEG	The most consistent and significant MT effect occurred for mu rhythm

## Data Availability

Data used to support the findings of this review are included within the article.
